# Small RNA sequencing reveals sex-related miRNAs in *Collichthys lucidus*

**DOI:** 10.3389/fgene.2022.955645

**Published:** 2022-08-26

**Authors:** Wei Song, Wu Gan, Zhengli Xie, Jia Chen, Lumin Wang

**Affiliations:** ^1^ East China Sea Fisheries Research Institute, Chinese Academy of Fishery Sciences, Shanghai, China; ^2^ Key Laboratory of Exploration and Utilization of Aquatic Genetic Resources, Ministry of Education, Shanghai Ocean University, Shanghai, China; ^3^ Fishery Machinery and Instrument Research Institute, Chinese Academy of Fishery Sciences, Shanghai, China; ^4^ State Key Laboratory of Large Yellow Croaker Breeding, Fuding Seagull Fishing Food Co. Ltd., Ningde, China

**Keywords:** *Collichthys lucidus*, small RNA sequencing, miR-430c-3p, *bmp15*, sex differentiation

## Abstract

*Collichthys lucidus* (*C. lucidus*) is an economically important fish species, exhibiting sexual dimorphism in its growth rate. However, there is a lack of research on its underlying sex-related mechanisms. Therefore, small RNA sequencing was performed to better comprehend these sex-related molecular mechanisms. In total, 171 differentially expressed miRNAs (DE-miRNAs) were identified between the ovaries and testes. Functional enrichment analysis revealed that the target genes of DE-miRNAs were considerably enriched in the p53 signaling, PI3K–Akt signaling, and TGF-beta signaling pathways. In addition, sex-related miRNAs were identified, and the expression of miR-430c-3p and miR-430f-3p was specifically observed in the gonads compared with other organs and their expression was markedly upregulated in the testes relative to the ovaries. *Bmp15* was a target of miR-430c-3p and was greatly expressed in the ovaries compared with the testes. Importantly, miR-430c-3p and *bmp15* co-expressed in the ovaries and testes. This research provides the first detailed miRNA profiles for *C. lucidus* concerning sex, likely laying the basis for further studies on sex differentiation in *C. lucidus*.

## Introduction

Spinyhead croaker (*Collichthys lucidus*, *C. lucidus*) is an economically significant fish species and is broadly spread in the coastal waters of the north-western Pacific Ocean, including around China and Japan (Cheng et al., 2012). *C. lucidus* shows sexual dimorphism in its growth rate, and the female is larger than the male (Cai et al., 2019; Lai et al., 2022). It feeds mainly on benthic organisms, little fish, and shrimps. The critical seasons of its development and reproduction are spring and summer. Because of the variation of water temperature in different sea areas, the spawning season of the fish differs in different areas (Li, 2019). At present, studies on *C. lucidus* mainly focus on feeding ecology, genetic diversity and phylogenetic analysis (Wang J. F. et al., 2016; Song et al., 2016; Zhao et al., 2021). Overfishing has resulted in a decline in *C. lucidus* population size, which makes it impossible to meet market demand (Sang et al., 2015). As a result, to ensure a stable *C. lucidus* population, it is essential to elucidate the molecular mechanisms underlying reproduction and sex differentiation in this species. A previous study revealed that 333 and 556 unigenes were specifically expressed in the ovaries and testes, respectively, via *de novo* transcriptome sequencing of *C. lucidus*, and *hyal*, *sycp3*, and *bmp15* might play an important role in reproduction and sex determination (Song et al., 2020). Furthermore, Li et al. (2019) illustrated that *vasa* was highly conserved in *C. lucidus* and may take part in germ cell development. But, to date, the underlying sex-related molecular mechanisms in *C. lucidus* remain unknown.

MicroRNAs (miRNAs) are a class of endogenous non-coding RNAs that are 18–25 nucleotides (nt) in length and are widely present in eukaryotes; post-transcriptionally, they function as negative modulators of gene expression by binding to the 3′-untranslated region (UTR) of the mRNA (Krol et al., 2010; Kumar, 2014). Previous research has shown that miRNAs control various biological processes, such as cell proliferation, cell differentiation, apoptosis, immune responses, and metabolism (Andreassen and Hoyheim, 2017; Herkenhoff et al., 2018; Zhou et al., 2018). Recently, miRNA expression levels in numerous fish species, including zebrafish, Nile tilapia, and medaka, were investigated using transcriptome sequencing (Lau et al., 2014; Pinhal et al., 2018; Ji et al., 2019). Researchers have reported the role of sex-biased miRNAs in teleosts (Bhat et al., 2020). Particularly, Zhang et al. (2017a) found that the expression of miR-202–5p was higher in zebrafish throughout oocyte maturation, implying its function in germ cell growth. Xiao et al. (2014) discovered that the miR-727, miR-129, and miR-29 families exhibited remarkably higher expression in the ovaries compared with that in the testes of Nile tilapia, signifying that miRNAs regulate gonadal development and reproduction. In addition, Qiu et al. (2018) studied miRNA expression in medaka gonads using RNA sequencing and showed that miR-202–5p, miR-430c, and miR-26a were expressed in a gonad-specific or sex-biased pattern. Wang et al. (2017) found that 431 and 628 miRNAs were specifically expressed in adult and juvenile ovaries in *Cyprinus carpio*, respectively, providing the basis for further research on the role of miRNAs in the modulation of ovarian differentiation. Together, these studies showed that miRNAs are involved in reproduction and sex differentiation. Furthermore, miRNAs can regulate reproduction and gonadal development by targeting several genes. For instance, miR-26 has been shown to regulate the expression level of *ddx3x* in medaka (Sun et al., 2020). In *Carassius auratus*, miR-430 could regulate primordial germ cell development by targeting C1q-like factor (Mei et al., 2013). However, there is little information regarding the role of miRNAs in *C. lucidus*.

In this study, we investigated the sex-related molecular mechanism using small RNA sequencing of ovaries and testes in *C. lucidus*. In addition, we identified differentially expressed miRNAs (DE-miRNAs) between the ovaries and testes, and analyzed their target genes based on differentially expressed mRNAs (DE-mRNAs) between the ovaries and testes from the *de novo* transcriptome findings of our previous research (Song et al., 2020). Furthermore, we identified the signaling pathways connected with sex. The findings of our study may lay a foundation for developing a comprehensive understanding of the molecular mechanism underlying the function of miRNAs in the regulation of sex differentiation in *C. lucidus*.

## Materials and methods

### Sample collection

Ten *C. lucidus* individuals (five females and five males) were collected from the East China Sea in Ningde, Fujian Province, China, during the breeding period from May to June. Males had a mean body length of 12.07 ± 1.25 cm and a mean body weight of 33.80 ± 10.75 g; females had a mean body length of 12.05 ± 1.50 cm and a mean body weight of 36.75 ± 13.04 g. The fish were anesthetized with tricaine methanesulfonate (100 mg/L) and positioned on ice for the dissection of organs, including the brain, eyes, gills, fins, stomach, liver, kidneys, intestines, heart, muscle, skin, ovaries, and testes. These samples were instantly frozen in liquid nitrogen and stored at −80°C before use.

This study was conducted according to the guidelines of the Declaration of Helsinki, and approved by the Animal Research and Ethics Committee of Shanghai Ocean University.

### Histological analysis of the gonads

The ovaries and testes were fixed in Bouin solution, paraffin-embedded, and horizontally and vertically cut into continuous sections with thicknesses of 6–8 μm. These sections were then subjected to hematoxylin and eosin (HE) staining. Using Image-Pro Plus 5.1 software, the diameter of the germ cells in each phase was examined. Additionally, the ovarian stage and the time phase division of oocytes were investigated based on the traditional fish oocyte development stage criteria; these data were combined with the experimental observation results. Ovarian development can be divided into six stages. The ovaries are translucent and threadlike in stage I, with hardly any visible blood vessels on the surface. The ovaries are dominated by the first phase oocytes. Phase I oocytes transit from oogonia to primary oocytes, and oocytes are triangular or erratically rounded. In stage II, the surface of the ovaries is mostly light yellow, and the ovaries are dominated by the second phase oocytes. Phase II oocytes are at the small growth phase of primary oocytes, and the oocytes are polygonal and round. In stage III, the ovaries are dilated, full of thick blood vessels and pale yellow, and eggs are noticeable to the naked eyes. Oocytes of stage III dominate stage III ovaries, and there are also a small number of oocytes of stage I and II. Phase III oocytes are at the grand phase of growth of primary oocytes, and the oocytes are fusiform. In stage IV, ovaries reach the largest, and are orange, full, and oval. Stage IV oocytes dominate stage IV ovaries, and there are still a small number of stage II and III oocytes. Phase IV oocytes are at the late phase of growth of primary oocytes, and oocytes are enlarged. In stage V, the ovaries are loose and swollen, with tremendously developed blood vessels. The eggs are transparent and free in the ovarian capsule. Stage V ovaries are dominated by the stage V oocytes. Phase V oocytes transit from primary oocytes to secondary oocytes, and the cytoplasm is uniformly covered with coarse yolk granules. In stage VI, the ovaries are congested and the follicular cells that have released the ovum grow larger. The definition of each stage of ovarian development was based on the proportion of oocytes occupying more than 50% of the ovarian section or those occupying the highest proportion of oocytes.

### RNA isolation, RNA library preparation, and small RNA sequencing

Total RNA was extracted from the ovary and testis of three females and three males of *C. lucidus* using the Trizol reagent (Invitrogen, Carlsbad, CA, United States) according to the manufacturer’s instructions. The NanoDrop 2000 spectrophotometer (Thermo, United States) and Agilent 2100 bioanalyzer (Agilent Technologies, CA, United States) were employed to assess the quality and purity of RNA. The RNA quality criteria were as follows: A260/A280 ratio >1.8 and RIN value ≥8.0. Purified RNA samples were performed for adapter ligation, cDNA synthesis and PCR amplification, and 8% SDS-PAGE gel for sorting of fragments to obtain miRNA libraries using NEBNext Multiplex Small RNA Library Prep Kit (New England Biolabs, United States) by reference to the manufacturer’s instruction. Lastly, all libraries were sequenced using HiSeq 2500 (Illumina, United States). All sequences have been deposited in the NCBI sequence read archive with accession number: PRJNA660860.

### Analysis of small RNA sequencing data

The raw reads were processed using Fast-QC (v0.11.4) by first filtering adaptor sequences and low-quality sequences (Q scores ≤10%, lengths <18 nt). Afterward, the remaining 18–32 nt reads were taken as clean reads. The Rfam database was used to annotate small RNAs by removing non-coding RNAs, such as rRNA, scRNA, snoRNA, snRNA, and tRNA. The clean reads were then mapped against the reference genome of *C. lucidus*. The reads were searched against precursor/mature miRNAs in miRBase 21.0 (http://www.mirbase.org/) to identify known miRNAs. To identify novel miRNAs, the secondary structures of the reads were predicted using RNAfold (http://rna.tbi.univie.ac.at/cgibin/RNAWebSuite/RNAfold.cgi).

### Analysis of DE-miRNAs and target gene prediction

DE-miRNAs were analyzed using the DE-Seq 2.0 algorithm, with significant differences for each miRNA determined according to ∣log2 fold change∣> 1 and false discovery rate <0.05. To understand the expression patterns of DE-miRNAs, hierarchical clustering of DE-miRNAs was performed using Cluster 3.0. The target genes of each DE-miRNA were predicted via the analysis of differentially expressed genes between the ovaries and testes from the results of previous *de novo* transcriptome sequencing (Song et al., 2020). In brief, the 3′-UTR sequences of *C. lucidus* mRNAs were determined by the results of the *de novo* transcriptomes. The putative 3′-UTR sequences of DE-mRNAs were used for predicting DE-miRNA–DEmRNAs using RNAhybrid (http://bibiserv.techfak.uni-bielefeld.de/cgi-bin/rnahybrid_tdb_mirnas.cgi) and miRanda (http://www.microrna.org/). The DE-miRNA–DEmRNA network was constructed using Cytoscape V3.6.0 (https://cytoscape.org/).

### Functional enrichment analysis

Gene Ontology (GO, http://www.geneontology.org) enrichment analysis and Kyoto Encyclopedia of Genes and Genomes (KEGG, http://www.genome.jp/kegg) pathway analysis were employed to identify the function of DE-miRNAs. The threshold for significance in GO and KEGG analyses was defined by a false discovery rate of <0.05.

### Validation of DE-miRNAs and *bmp15*

The expression levels of the five DE-miRNAs (miR-430c-3p, miR-430f-3p, miR-22b-3p, miR-222a-5p, and miR-222b-5p) and *bmp15* were validated via quantitative reverse transcription-polymerase chain reaction (qRT-PCR). These five DE-miRNAs were selected because they were involved in Sex differention and development function and had relatively high fold change compared with other miRNAs in the miRNA-mRNA-Pathway network. *Bmp15* was selected since it was a target of miR-430c-3p. Total RNA from 13 organs, including testis, ovary, brain, eyes, gills, pectoral fins, heart, liver, stomach, renal, intestinal tract, muscle, and skin from three females and three males of *C. lucidus* was extracted using the Trizol reagent (Invitrogen, Carlsbad, CA, United States). Then, first-strand cDNA was synthesized using the PrimeScript RT Kit (Takara, Dalian, China). Subsequently, qRT-PCR was conducted using the ABI QuantStudio 6 Flex system (Applied Biosystems, Foster City, CA, United States) and the SYBR-Green PCR Kit (Roche Diagnostics, Indianapolis, IN, United States). The qRT-PCR cycling conditions were as follows: 95°C for 10 min, 45 cycles of 95°C for 15 s, 60°C for 60 s, and 95°C for 15 s. The relative miRNA expression was determined by adopting the 2^−ΔΔCt^ method. The U6 gene was used as an internal control for the five DE-miRNAs, and GAPDH was used as an internal control for *bmp15*. Each sample was analyzed in triplicate. The primer sequences used for qRT-PCR are presented in Supplementary Table S1.

### Fluorescence *in situ* hybridization (FISH)

Cy3-labeled miR-430c-3p probes (5′-CTA​CCC​CAA​AGA​GAA​GCA​CTT​A-3′) and Dig-labeled *bmp15* probes (5′-CGA​CGG​TTT​CAG​CAG​ACG​CAC-3′) were designed and synthesized by Genebio (Shanghai, China) and the probes’ sequences were available upon request. Briefly, paraffin sections were de-paraffinized and rehydrated through graded alcohols, and blocked endogenous peroxidase activity for 10 min at room temperature using 3% hydrogen peroxide and washed two times using PBS. The sections were added 3% pepsin at room temperature for 10 min, and incubated with pre-hybridization buffer for 4 h at 37°C prior to additional probes in hybridization buffer (BOSTER) at 37°C overnight. Subsequently, the samples were washed with 2× saline-sodium citrate (SSC) for 5 min, 0.5× SSC (BOSTER) for 15 min and 0.2× SSC for 5 min at 37°C. After blocking with 5% BSA in PBS for 30 min, the samples were incubated with secondary antibodies for 120 min at 37°C, and then stained with DAPI (Solarbio, Beijing, China) for 2 min. The tissue sections were observed under a fluorescent illumination microscope (Olympus IX71, Tokyo, Japan).

### Statistical analysis

Data were evaluated using SPSS 21.0 (IBM, Chicago, United States) and are presented as the mean ± standard deviation of three replicates. Hierarchical clustering of DE-miRNAs was carried out using Cluster 3.0. Differences between the different groups were compared using one-way ANOVA. Tukey’s HSD post hoc test was employed to determine differences detected by ANOVA. The differences were considered statistically significant at *p* < 0.05.

## Results

### Histological characteristics of the gonads in *C. lucidus*

**FIGURE 1:**
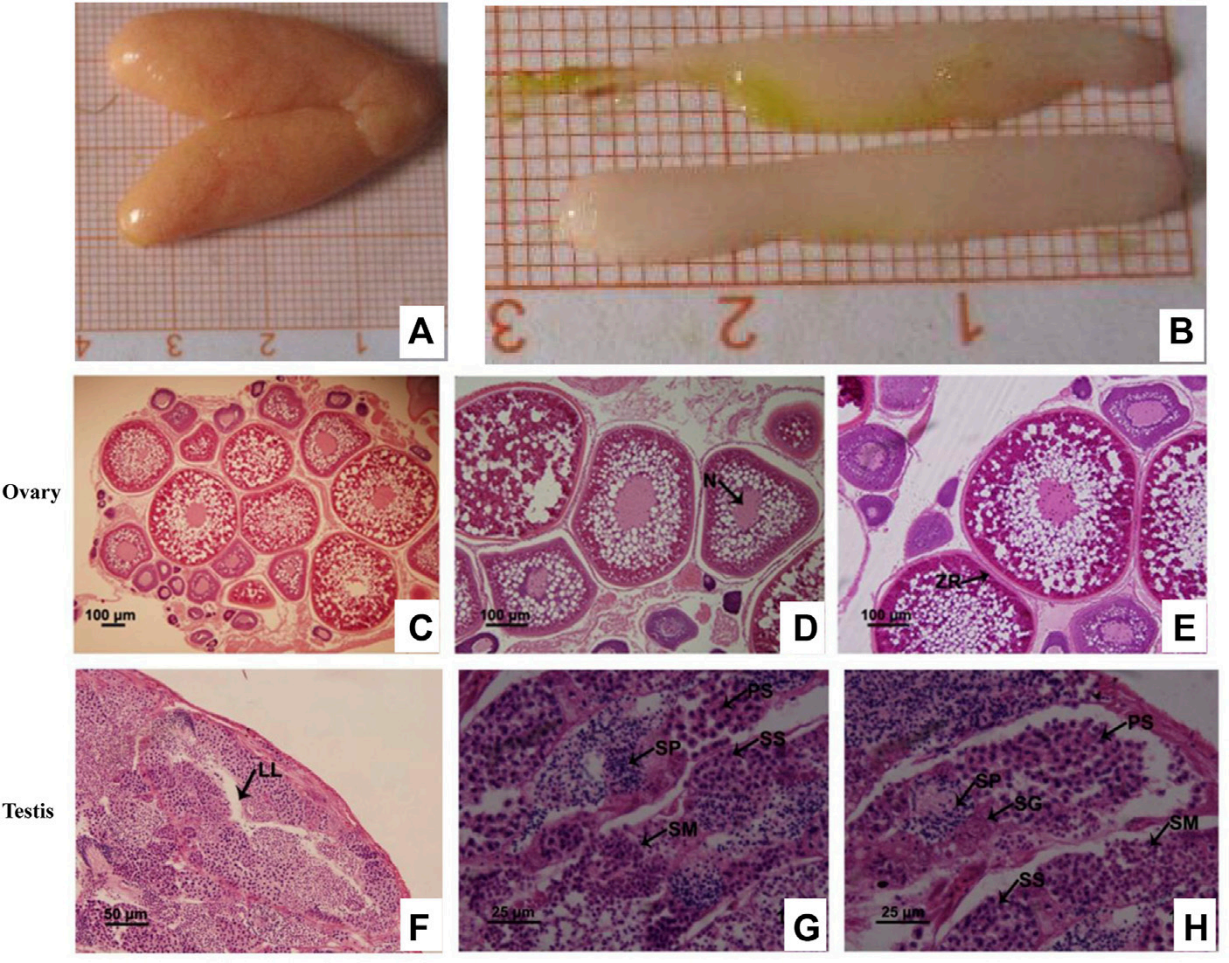
Histological characteristics show the specific periods of cells in the ovaries and testes from sexually mature *Collichthys lucidus*. **(A)** The morphology of the ovaries. **(B)** The morphology of the testes. **(C)** Hematoxylin and eosin (HE) staining of the ovary at stage IV. **(D)** Early oocytes in phase IV. Arrow shows the deformed nuclei (N). **(E)** Metaphase oocytes in phase IV. Arrow shows the zona radiate (ZR). **(F)** HE staining of the testis at stage IV. Arrow shows the lobular lumen (LL). **(G,H)** Germ cells in the testis at stage IV. PS: primary spermatocyte; SG: spermatogonia; SM: spermatid; SP: spermatozoa; SS: secondary spermatocyte. Note: Figure 1D and Figure 1E are specific periods of oocytes in stage IV ovary. Figure 1G and Figure 1H are specific periods of germ cells in stage IV testis. Different images show different cells at a specific period, and the images are from a field of the same sample, so there is overlap view between the images to illustrate different cells.

HE staining revealed that during the breeding season (i.e., between May and June), most ovaries and testes of *C. lucidus* had entered the mature stage and were primarily in stage IV. Stage IV ovaries reached the maximum with 24.0–56.0 mm in length and 8.1–20.7 mm in width, and were orange, full and oval (Figure 1A). In stage IV ovaries, phase IV oocytes were the predominant cells, although there was still a small number of phase II and III oocytes (Figure 1C). Phase IV cells were primary oocytes that occurred at the later stage of development, with an enlarged size and thickened radiation belt. Yolk granules almost filled the outer space of the nucleus and the cytoplasm appeared only around the nucleus and near the edge of the cytomembrane (Figures 1D,E). The testes of *C. lucidus* were the radiate type; the fine lobules were arranged in a regular radial pattern. The lumen, through which the sperm is expelled, was in the center of the spermatozoa (Figure 1F). Stage IV testes were full, opalescent, rod-shaped, blunt at the front end, tapered at the back end, 17.1–38.3 mm in length, and 3.2–7.9 mm in width (Figure 1B). The lobular lumen was unexpanded and mainly contained the spermatid, secondary spermatocytes, spermatozoa, primary spermatocytes, and spermatogonia (Figure 1G). Compared with spermatogonial cells, primary spermatocytes were smaller in size, had a sparse arrangement and were round or oval in shape (Figure 1H).

### High-throughput small RNA sequencing

**FIGURE 2:**
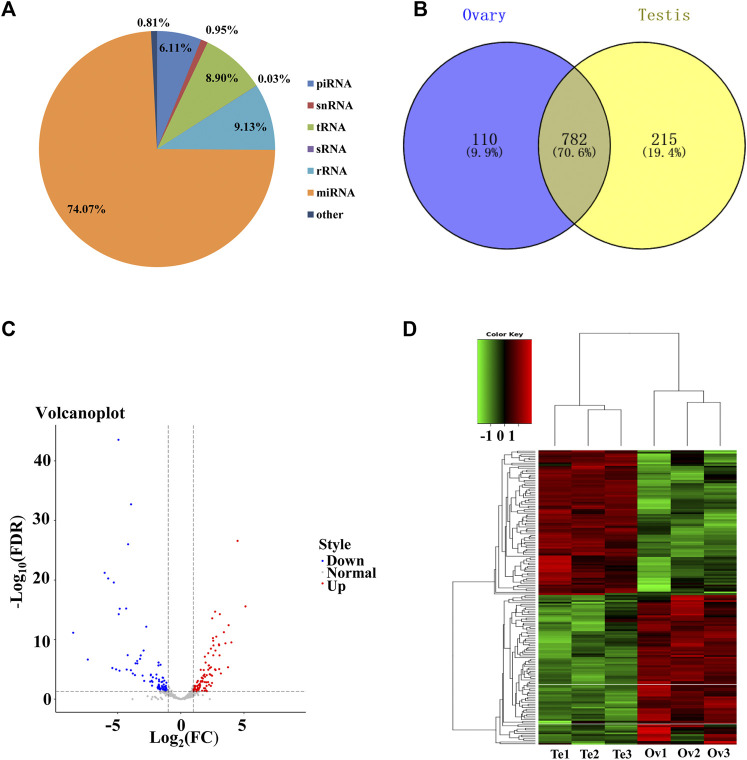
Genomic profiling of the miRNAs in the gonads of *Collichthys lucidus*. **(A)** The proportion of the different types of RNAs annotated by the Rfam database. **(B)** Venn diagram showed the unique and common miRNAs identified in the ovaries and testes. **(C)** Volcano plot of the DE-miRNAs between the ovaries and testes of *C. lucidus*. The red and blue dots represent up- and downregulated DE-miRNAs in the ovaries, respectively. The gray dots represent the miRNAs without significantly differential expression. **(D)** Hierarchical clustering of the DE-miRNAs between the ovaries and testes of *C. lucidus*. Te1, Te2, and Te3 represent testis samples; Ov1, Ov2, and Ov3 represent ovary samples. The heatmap colors represent relative miRNA abundance: red and blue indicate high and low miRNA expressions, respectively.

To identify the sex-related miRNAs in *C. lucidus*, small RNA libraries from ovaries and testes were constructed and sequenced. A total of 34,111,577 and 36,919,273 raw reads were generated from the ovaries and testes, respectively. After removing low-quality sequences and adaptor contaminants, 32,160,826 and 35,165,432 clean reads were obtained from the ovaries and testes, respectively (Supplementary Table S2). The clean reads were mapped against the reference genome of *C. lucidus*, and the number of mapped clean reads ranged from 21,934,375 to 37,724,397, with a mapping rate of 76.8%–80.6% (Supplementary Table S3). The length distribution of the unique small RNA reads was 18–32 nt, and two peaks occurred at lengths of 21–23 nt and 26–29 nt (Supplementary Figure S1). The small RNAs were mapped to the Rfam database (Figure 2A). Among all the matched reads, more than 31.25 million reads (74.07%) were annotated as miRNAs, which were the most abundant RNA type, followed by rRNAs (9.13%), tRNAs (8.90%), piRNA (6.11%), snRNAs (0.95%) and sRNA (0.03%). After removing the miRNAs represented by less than 10 reads in all samples, a final set of 1,107 miRNAs was identified in *C. lucidus* (Figure 2B)*.* Interestingly, 782 (70.6%) miRNAs were shared between the ovaries and testes, whereas 110 (9.9%) and 215 (19.4%) miRNAs were uniquely expressed in the ovaries and testes, respectively.

### DE-miRNAs between the ovaries and testes

In total, 171 DE-miRNAs were identified between the ovaries and testes of *C. lucidus* (Figure 2C). Among these, 87 and 84 DE-miRNAs were upregulated and downregulated in the ovaries, respectively, compared with the testes. Hierarchical cluster analysis indicated that these DE-miRNAs allowed the separation of the samples into ovary and testis groups (Figure 2D), validating the reliability of our analysis. The top 5 upregulated and downregulated miRNAs with large expression changes were determined. As shown in Table 1, miR-21a-2-3p, miR-223–3p, miR-489–5p, miR-18b-5p and miR-489–3p were significantly upregulated in the ovary compared with the testis. However, miR-430c-3p, miR-212–3p, miR-212a-5p, miR-724–5p, and miR-132–3p were remarkably upregulated in the testis compared with the ovary.

**TABLE 1:** The expression of DE-miRNAs in the ovary and testis of *Collichthys lucidus*. Ten DE-miRNAs with largest expression changes were identified in the ovary and testis.

AccID	log2FC	Pvalue	Ov-1	Ov-2	Ov-3	Te-1	Te-2	Te-3
miR-21a-2-3p	5.124632457	5.64E-18	228	285	195	10	8	1
miR-223–3p	4.497612052	2.15E-29	6349	4758	5783	251	223	215
miR-489–5p	4.008176818	1.74E-11	223	896	343	26	42	10
miR-18b-5p	3.788526066	1.51E-14	605	368	513	31	32	36
miR-489–3p	3.7418984	5.53E-07	30	170	70	6	9	2
miR-430c-3p	−8.51650839	3.34E-13	1	1	0	119	240	261
miR-212–3p	−7.361163412	2.17E-08	0	0	1	47	68	43
miR-212a-5p	−6.026767369	8.27E-24	2	5	4	231	246	157
miR-724–5p	−5.74810811	8.74E-23	173	379	1068	27,861	28,834	18,726
miR-132–3p	−5.302915537	4.87E-22	3	8	10	202	307	206

### Target gene analysis of DE-miRNAs

**FIGURE 3:**
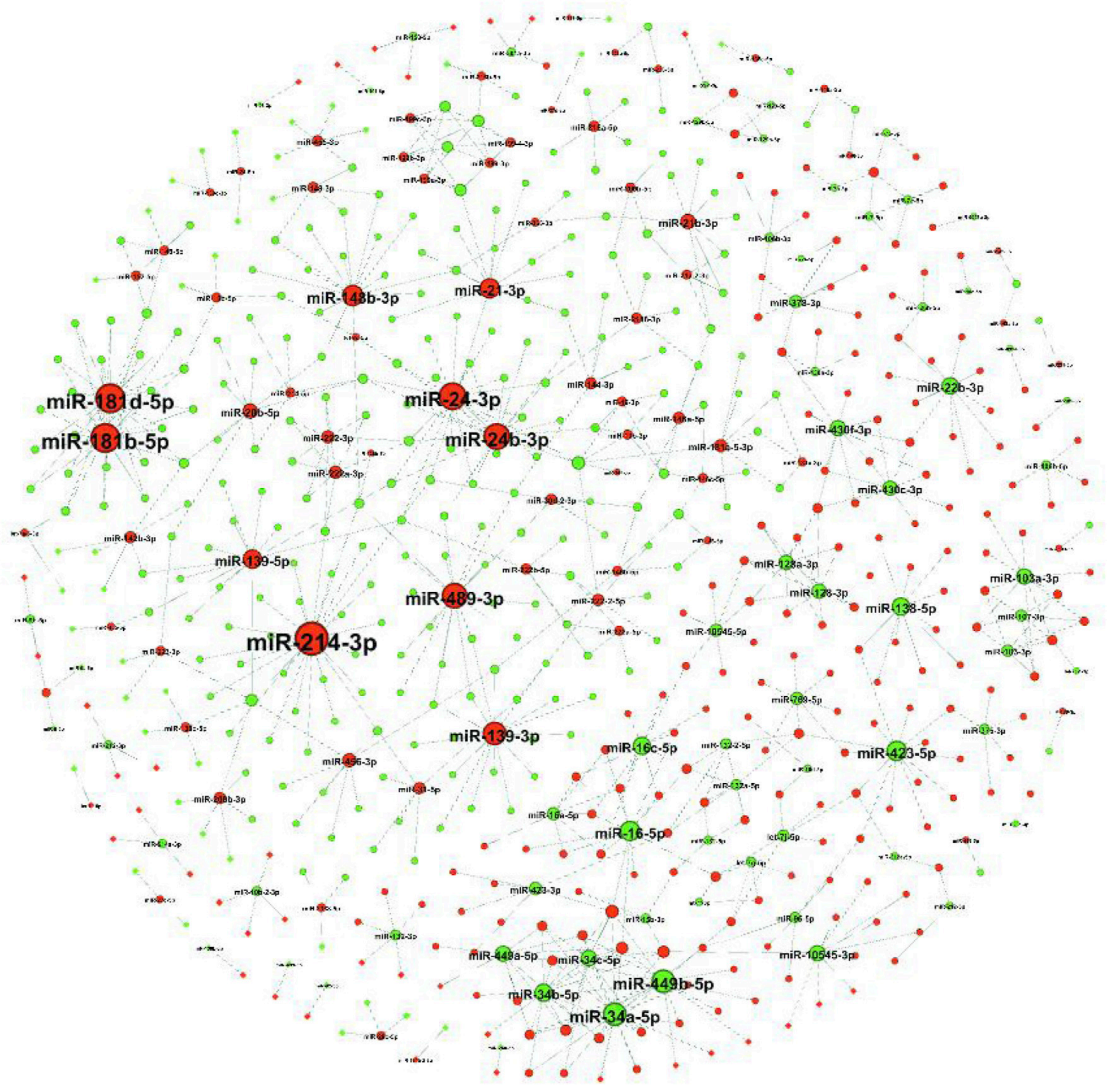
Target gene predictions of DE-miRNAs. A regulatory network of DE-miRNAs and their target genes that were negatively regulated. Nodes represent the miRNAs or mRNAs (here only the miRNA symbol is shown). The red and blue nodes represent up- and downregulated RNAs in the ovaries and testis, respectively.

The target genes of DE-miRNAs were predicted using RNAhybrid and miRanda, and using DE-mRNAs between the ovaries and testes from previous *de novo* transcriptome results (Song et al., 2020). A total of 423 up-miRNA−down-mRNA pairs and 349 down-miRNA−up-mRNA pairs were obtained, and 485 DE-mRNAs were predictively targeted by 144 DE-miRNAs (Figure 3). The upregulated miR-214–3p was predictively bound to the largest number of target genes (26 target genes), followed by the upregulated miR-181b-5p and miR-181d-5p (22 target genes each).

### Functions and pathways of DE-miRNAs

**FIGURE 4:**
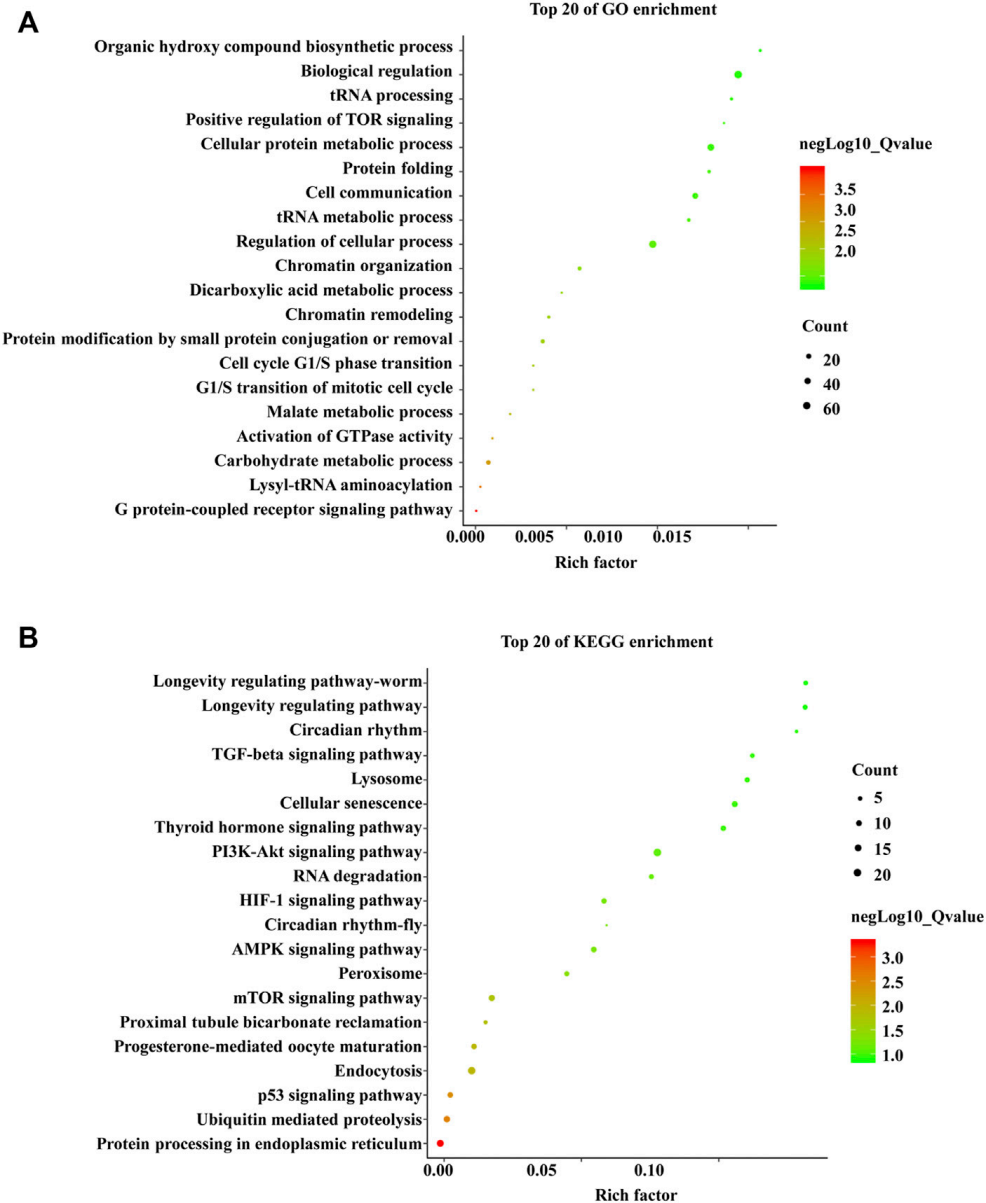
Gene Ontology (GO) and Kyoto Encyclopedia of Genes and Genomes (KEGG) enrichment analyses of the DE-miRNAs in the gonads of *Collichthys lucidus*. **(A)** GO enrichment analysis of the DE-mRNAs targeted by DE-miRNAs. The *Y*-axis represents GO terms and the *X*-axis represents the rich factor. The dot size represents the number of DE-mRNAs enriched in the GO terms, and the color represents the significance of the GO term. **(B)** KEGG enrichment analysis of the DE-mRNAs targeted by DE-miRNAs. The figure shows the 20 most enriched pathways between the ovaries and testes. The *Y*-axis represents pathways and the *X*-axis represents the rich factor. The dot size represents the number of DE-mRNAs enriched in the pathway, and the color represents the significance of the pathway.

To explore the biochemical functions and signaling pathways of the DE-miRNAs between the ovaries and testes, GO and KEGG enrichment analyses of the target genes of DE-miRNAs were conducted. GO analysis revealed that the target genes were mainly involved in the G-protein-coupled receptor signaling pathway, lysyl-tRNA aminoacylation, carbohydrate metabolic process, and activation of GTPase activity (Figure 4A). KEGG analysis revealed that the target genes were mostly associated with the p53 signaling pathway, AMPK signaling pathway, PI3K–Akt signaling pathway, TGF-beta signaling pathway, and progesterone-mediated oocyte maturation (Figure 4B).

### Analysis of sex-related DE-miRNAs

**FIGURE 5:**
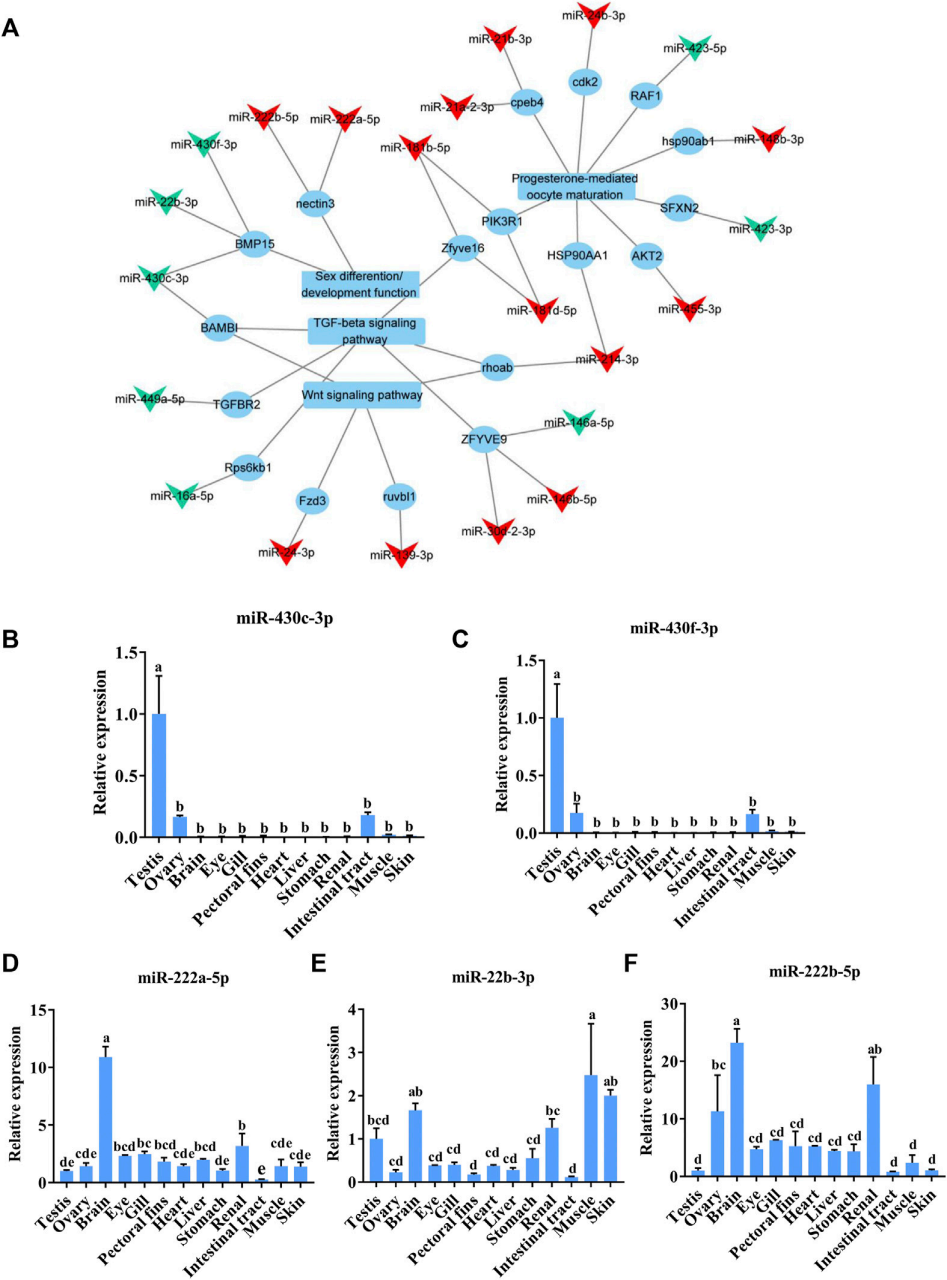
Analysis of sex-related DE-miRNAs in *Collichthys lucidus*. **(A)** The regulatory network of DE-miRNAs and sex-related genes, functions, and pathways. The triangular nodes represent miRNAs, the circular nodes represent mRNAs, and the quadrangular nodes represent functions and pathways. The red and green nodes represent up- and downregulated RNAs in the ovary compared to those in the testis, respectively. **(B–F)** The expression of sex-related candidate miRNAs was verified via qRT-PCR in various *C. lucidus* organs. Three biological replicates were performed for each experiment. One-way ANOVA with Tukey’s HSD post hoc test. The difference is insignificant if there is one marked with the same lowercase letter (*p* > 0.05), and the difference is significant if there is a different marked lowercase letter (*p* < 0.05).

To identify the potential sex-related miRNAs, we analyzed the DE-miRNAs targeting sex-associated genes, functions, or pathways, including progesterone-mediated oocyte maturation, the TGF-beta signaling pathway, and the Wnt signaling pathway, which are known to be involved in sex determination and differentiation in other fish species (Herpin et al., 2013; Depiereux et al., 2015; Jia et al., 2018). After establishing a DE-miRNA-mRNA-sex-related pathway network, we found that 22 DE-miRNAs potentially regulated the sex-related pathways by targeting 18 mRNAs (Figure 5A). Interestingly, miR-430c-3p, miR-430f-3p, and miR-22b-3p targeted *bmp15*, which is known to be involved in sex determination in *C. lucidus* (Song et al., 2020). In addition, miR-222b-5p and miR-222a-5p were predicted to target *nectin3*, which plays a major role in mouse spermatid development (Herpin et al., 2013). The five aforementioned DE-miRNAs—namely miR-430c-3p, miR-430f-3p, miR-22b-3p, miR-222a-5p, and miR-222b-5p—were chosen for qRT-PCR verification based on the above results in addition to their relatively high fold change compared with other miRNAs in the network. The expression trend of these miRNAs through qRT-PCR was in accordance with the RNA-seq results (Figures 5B–F). Interestingly, miR-430c-3p and miR-430f-3p were specifically expressed in the gonads, whereas they were significantly upregulated in the testes compared with the ovaries. Additionally, miR-22b-3p, miR-222a-5p, and miR-222b-5p were broadly expressed in all the *C. lucidus* organs.

### MiR-430c-3p and *bmp15* co-express in the ovaries and testes

**FIGURE 6:**
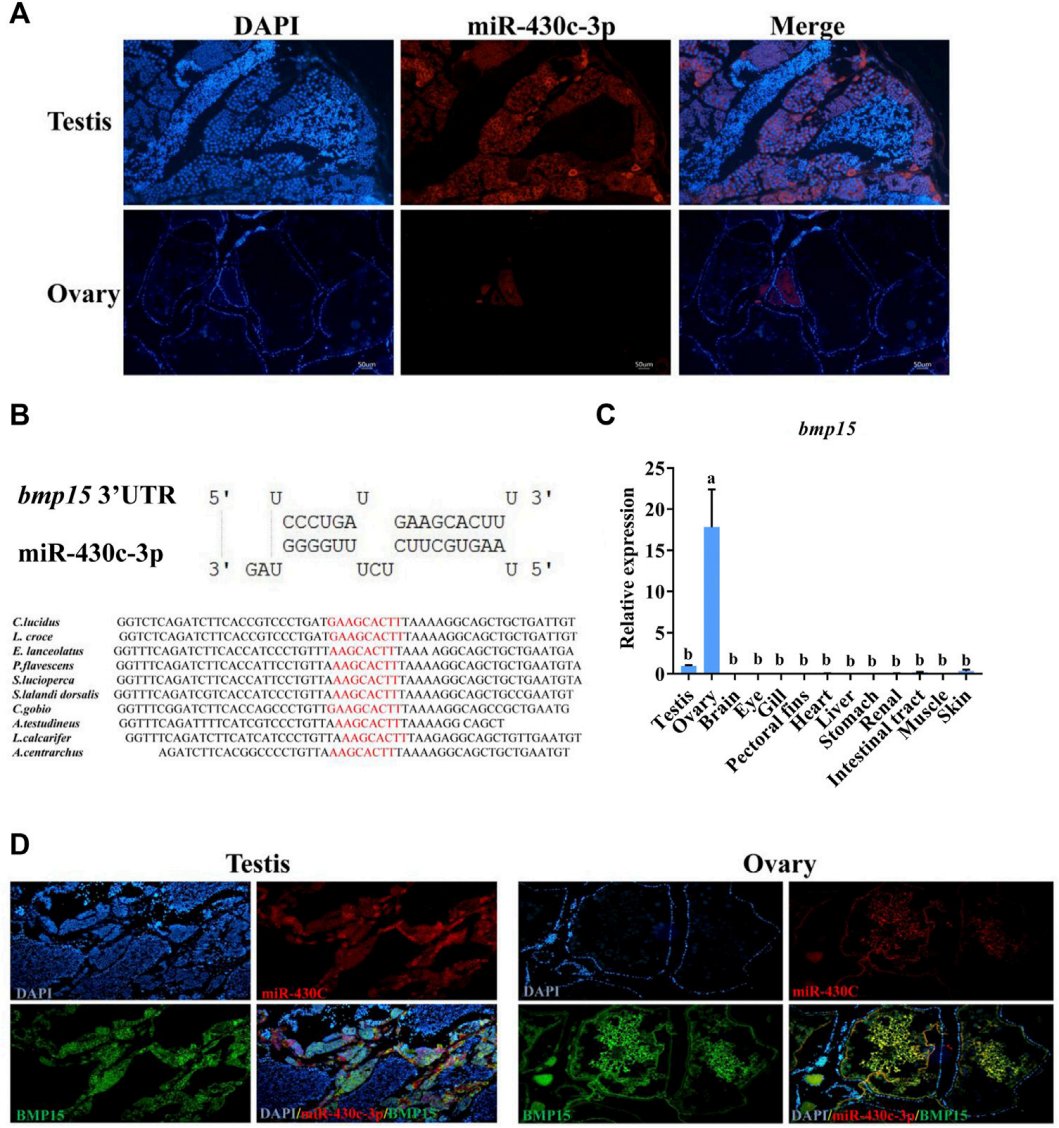
MiR-430c-3p and *bmp15* co-express in the ovaries and testes. **(A)** Fluorescence *in situ* hybridization (FISH) showed that miR-430c-3p was predominantly localized in the testes. MiR-430c-3p probes were labeled with cy3 (red), and nuclei were stained with DAPI (blue). **(B)** The binding sequences between miR-430c-3p and *bmp15* were predicted by RNAhybrid and miRanda and their sequences were analyzed for conservation. **(C)** The expression of *bmp15* was verified via qRT-PCR in various *C. lucidus* organs. N = 3. One-way ANOVA with Tukey’s HSD post hoc test. The difference is insignificant if there is one marked with the same lowercase letter (*p* > 0.05), and the difference is significant if there is a different marked lowercase letter (*p* < 0.05). **(D)** FISH showed co-localization between miR-430c-3p and *bmp15* in ovaries and testes. *Bmp15* probes were labeled with dig (green). MiR-430c-3p probes were labeled with cy3 (red).

Based on the qRT-PCR results, we selected the miR-430c-3p to further identify expression in gonads using FISH. The expression of miR-430c-3p was in agreement with qRT-PCR results (Figure 6A). Subsequently, RNAhybrid and miRanda identified *bmp15* as a potential target of miR-430c-3p, as the 3′UTR of fish *bmp15* mRNA contains potential binding sequences which was conserved in teleosts (Figure 6B). The *bmp15* was aslo specifically expressed in the gonads, and was highly expressed in the ovaries compared with in the testes (Figure 6C). To determine interdependent role of miR-430c-3p and *bmp15*, we further detected whether miR-430c-3p and *bmp15* were colocalized in the ovaries and testes by FISH (Figure 6D). The result showed that miR-430c-3p co-expressed with *bmp15* in the ovaries and testes, whereas their fluorescence intensity was inversed.

## Discussion

*C. lucidus* is an important economical fish; however, there is a lack of research on sex differentiation in *C. lucidus*. Small RNA sequencing has been applied in numerous aquatic species, including medaka, tilapia, and zebrafish (Vaz et al., 2015; Wang B. et al., 2016; Qiu et al., 2018). In this study, small RNA sequencing was performed on the ovary and testis of *C. lucidus* to screen sex-related miRNAs. To the best of our understanding, this study was the first attempt to perform small RNA sequencing in *C. lucidus*, which provided a molecular basis for understanding of the gonadal development and sex differentiation of this species. In the present study, we identified 171 DE-miRNAs between the ovaries and testes, which can lay a foundation for further study on the molecular mechanism by which miRNAs regulate sex differentiation in *C. lucidus*.

In the present study, the target genes of DE-miRNAs were mainly involved in the p53 signaling, PI3K-Akt signaling, and TGF-beta signaling pathways. The p53 signaling pathway was previously found to be enriched in the ovaries of *Oplegnathus punctatus* (Du et al., 2017). Another study has reported that this pathway broadly participates in folliculogenesis and oogenesis (Hussein, 2005). In zebrafish, the p53 signaling pathway can regulate sex differentiation through the gonadal transcriptome (Ribas et al., 2017). Jia et al. (2018) discovered that differentially expressed genes were connected with the TGF-beta signaling pathway during ovarian development in the Yellow River carp (*Cyprinus carpio*). The transcription factor *smad4*, the main component of the TGF-beta signaling pathway, has been discovered to be highly expressed in the ovaries of carp. *Gsdf* and *amh*, two members of the TGF-beta subfamily, have been discovered to be prominently expressed in the testes, in contrast with the ovaries, in *Acipenser schrenckii* (Zhang et al., 2020). Moreover, Wu et al. (2015) observed higher expression levels of the PI3K–Akt signaling pathway genes in male rather than in female yellow catfish, whereas Zhang et al. (2017b) discovered that the activation of this pathway regulated sperm motility in yellow catfish. These results indicate that the PI3K–Akt signaling pathway plays critical roles in testicular development and spermatogenesis in yellow catfish. Considering these findings and our results, we hypothesize that the p53 signaling, PI3K–Akt signaling, and TGF-beta signaling pathways are involved in sex differentiation and gonadal development in *C. lucidus*.

Hypothalamus-pituitary-gonad (HPG) axis plays a major role in fish reproduction. A variety of steroid hormones including steroid hormones and gonadotropin hormones in the HPG axis can regulate gonadal development (Jin et al., 2016; Du et al., 2019). A previous study suggested that kisspeptin 2 enhances the expression levels of *gnrh* and *gth* genes in HPG axis in Nile tilapia and modulate gonadal maturation (Park et al., 2016). Another research discovered that pyriproxyfen can modulate reproductive endocrine system by regulating the expression of HPG axis genes in zebrafish (Maharajan et al., 2020). Therefore, in this study, we detected the expression of miRNA in 13 organs, including testis, ovary and brain. Numerous miRNAs are connected with gonadal development and sex determination in fish species, including rainbow trout, zebrafish, and Nile tilapia (Juanchich et al., 2013; Wang W. et al., 2016; Wong et al., 2018). MiR-200 family members indicated the relative low expression in YY testis and it revealed that miR-200 plays important function during testis development and spermatogenesis in yellow catfish (Jing et al., 2014). In tilapia, miR-143 showed high expression in the testis, indicating that it may participate in the development of testes (Xiao et al., 2014). MiR-101a showed abundant expression in the ovary of *Cyprinus carpio*, demonstrating a role of miR-101a in gonad development (Wang et al., 2017). MiR-202 was found to impact follicular recruitment and growth in medaka, thus regulating the female reproduction (Gay et al., 2018). Overall, these studies suggest that miRNAs play important roles in gonadal development and reproduction. In this study, miR-430c-3p and miR-430f-3p were mainly expressed in the ovaries and testes. The miR-430 family is known to be involved in gonadal development and primordial germ cell migration (Tani et al., 2010). MiR-430 has been reported to regulate primordial germ cell development in *C. auratus* and zebrafish (Mishima et al., 2006; Mei et al., 2013). MiR-430 also plays a vital role in ovarian differentiation and development in Yellow River carp (Wang et al., 2017). In medaka, miR-430c is significantly expressed in the ovaries compared with the testes (Qiu et al., 2018), which is in agreement with our findings. Additionally, miR-222 was discovered to play a vital role in ovarian development, and female reproduction (Zhu et al., 2019). Similarly, in the present study, miR-222a-5p and miR-222b-5p were highly expressed in the ovary of *C. lucidus*, signifying that they might regulate the ovarian development. In a previous study, miR-22b-3p was discovered to be associated with gonadal phenotypes in mammals (Ikert and Craig, 2020). In our study, miR-22b-3p was remarkably upregulated in the testes compared with the ovaries in our study, suggesting that miR-22b-3p may be related to the male development. Collectively, our results suggest that miR-430c-3p, miR-430f-3p, miR-222a-5p, miR-222b-5p, and miR-22b-3p may play important roles in the gonadal development in *C. lucidus*.

Predicting miRNA target genes is necessary for understanding the molecular mechanism underlying sex differentiation regulation. A study has shown that differentially expressed genes can be targeted by multiple miRNAs and DE-miRNAs may simultaneously regulate multiple genes (Kaufman et al., 2001). In this study, *bmp15* was discovered to be a common target gene of miR-430c-3p, miR-430f-3p, and miR-22b-3p. *Bmp15* is a member of the subgroup of the transforming growth factor β superfamily and can regulate ovarian development (Tan et al., 2009). In mice, *bmp15* has been found to impact oocyte development (Su et al., 2008). In sheep, *bmp15* is essential for normal folliculogenesis (Silva et al., 2011). In zebrafish, *bmp15* participates in the maintenance of adult female sex differentiation (Dranow et al., 2016). In a previous *C. lucidus* study, *bmp15* was remarkably upregulated in the ovaries compared with the testes (Song et al., 2020). Since only miR-430c-3p and miR-430f-3p were specially expressed in the gonads, and miR-430c-3p expression had relatively high fold change compared with miR-430f-3p, we paid attention to miR-430c-3p and *bmp15* in this study. MiR-430c-3p was discovered to co-express with *bmp15* in the ovaries and testes, whereas their expression was inversed. As a result, we postulate that miR-430c-3p takes part in sex differentiation by down-regulating *bmp15* in *C. lucidus*. This can assist us further research on the molecular mechanism of miR-430c-3p in sex differentiation and lay the basis for breeding trials of sex regulation in *C. lucidus*.

## Conclusion

This is the first research to analyze the sex-related miRNAs by small RNA sequencing. A total of 171 DE-miRNAs were identified between the ovaries and testes. Interestingly, miR-430c-3p and miR-430f-3p, which targeted *bmp15*, were specifically expressed in the gonads. MiR-430c-3p and *bmp15* co-expressed in the ovaries and testes. Our findings provide a foundation for future studies on sex differentiation in *C. lucidus*.

## Data Availability

The datasets presented in this study can be found in online repositories. The names of the repository/repositories and accession number(s) can be found in the article/supplementary material.
