# Supplementary material for: Small RNA sequencing reveals sex-related miRNAs in Collichthys lucidus
Source: Front Genet. 2022 Aug 26;13:955645. doi: 10.3389/fgene.2022.955645 (PMC9458855; doi:10.3389/fgene.2022.955645)
Supplement: Supplementary file 1 [file Table1.DOCX]

**Table S1 Information on the primers used in this study**

| **Name** | **Primer sequence (5’ to 3’)** |
| --- | --- |
| miR-430c-3p | RT:GTCGTATCCAGTGCGTGTCGTGGAGTCGGCAATTGCACTGGATACGACCTACCCC |
|  | F:CGCAGTAAGTGCTTCTCTTTG |
| miR-430f-3p | RT:GTCGTATCCAGTGCGTGTCGTGGAGTCGGCAATTGCACTGGATACGACCAACCCC |
|  | F:CGCAGTAAGTGCTTCTCTTTG |
| miR-22b-3p | RT:GTCGTATCCAGTGCGTGTCGTGGAGTCGGCAATTGCACTGGATACGACTCAGCTC |
|  | F:AAGCTGCCAGTTGAAGAG |
| miR-222a-5p | RT:GTCGTATCCAGTGCGTGTCGTGGAGTCGGCAATTGCACTGGATACGACGATCTAC |
|  | F:GCGCTCAGTAGTCAGTGTAG |
| miR-222b-5p | RT:GTCGTATCCAGTGCGTGTCGTGGAGTCGGCAATTGCACTGGATACGACGATCTAC |
|  | F:GTGCTCAGTAGTCAGTGTAG |
| All | R:AGTGCGTGTCGTGGAGTCG |
| U6 | F: CGATACAGAGAAGATTAGCATGGC |
| *bmp15*  GAPDH | R: AACGCTTCACGAATTTGCGT  F: CCCTCTGACTCCATCGTCTC  R: GGAATGGTAGCCGTAGTGGA  F: TCCATGCATACACAGCCACC  R: GCTTACCGTTGAGTTCAGGG |
