# Supplementary material for: Small RNA sequencing reveals sex-related miRNAs in Collichthys lucidus
Source: Front Genet. 2022 Aug 26;13:955645. doi: 10.3389/fgene.2022.955645 (PMC9458855; doi:10.3389/fgene.2022.955645)
Supplement: Supplementary file 2 [file Table3.DOC]

**Table S3 Statistic of small RNA mapping rate**

| Sample | Ovary-1 | Ovary-2 | Ovary-3 | Testis-1 | Testis-2 | Testis-3 |
| --- | --- | --- | --- | --- | --- | --- |
| Total | 28568153 | 32033571 | 34213672 | 36036820 | 48652722 | 35497707 |
| UnMapped | 6633778 | 6447751 | 7450270 | 7208686 | 10928325 | 6900528 |
| Mapped | 21934375 | 25585820 | 26763402 | 28828134 | 37724397 | 28597179 |
| MappedRate | 0.768 | 0.799 | 0.782 | 0.8 | 0.775 | 0.806 |
| UniqueMapped | 7410027 | 11773135 | 9038611 | 9998708 | 12962598 | 9483232 |
| UniqueMappedRate | 0.259 | 0.368 | 0.264 | 0.277 | 0.266 | 0.267 |
| RepeatMapped | 14524348 | 13812685 | 17724791 | 18829426 | 24761799 | 19113947 |
| JunctionAllMapped | 18742 | 14617 | 22287 | 46766 | 39808 | 41980 |
| JunctionUniqueMapped | 8085 | 8596 | 11399 | 12333 | 17297 | 11637 |
| AllBase | 7.48E+08 | 8.04E+08 | 9.06E+08 | 9.25E+08 | 1.25E+09 | 9.11E+08 |
| UnMappedBase | 1.66E+08 | 1.55E+08 | 1.89E+08 | 1.77E+08 | 2.72E+08 | 1.68E+08 |
| MappedBase | 5.82E+08 | 6.48E+08 | 7.17E+08 | 7.48E+08 | 9.74E+08 | 7.43E+08 |
| UniqueMappedBase | 1.92E+08 | 2.83E+08 | 2.4E+08 | 2.58E+08 | 3.3E+08 | 2.46E+08 |
| RepeatMappedBase | 3.9E+08 | 3.65E+08 | 4.77E+08 | 4.9E+08 | 6.44E+08 | 4.97E+08 |
