# Supplementary figures and images for: Small RNA sequencing reveals sex-related miRNAs in Collichthys lucidus
Source: Front Genet. 2022 Aug 26;13:955645. doi: 10.3389/fgene.2022.955645 (PMC9458855; doi:10.3389/fgene.2022.955645)

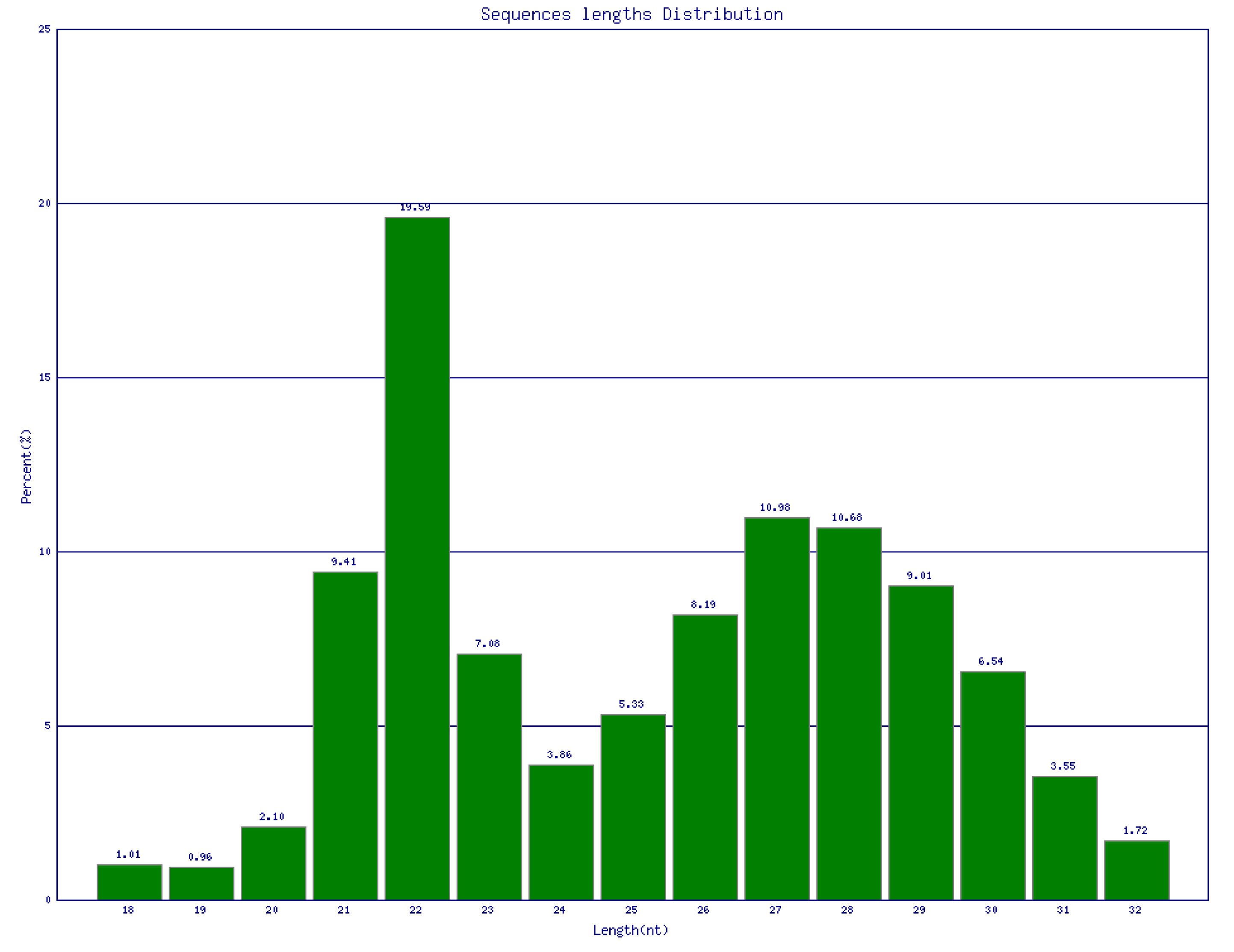

Supplement: Supplementary file 3 [file Image1.TIF]
