# Supplementary material for: Small RNA sequencing reveals sex-related miRNAs in Collichthys lucidus
Source: Front Genet. 2022 Aug 26;13:955645. doi: 10.3389/fgene.2022.955645 (PMC9458855; doi:10.3389/fgene.2022.955645)
Supplement: Supplementary file 4 [file Table2.DOCX]

**Table S2 Statistics of raw reads and clean reads in the ovary and testis of *C. lucidus***

| Sample | Raw_Reads | Clean reads | Q20% | Q30% | GC% |
| --- | --- | --- | --- | --- | --- |
| Ovary-1 | 30921490 | 29043700 | 77.37 | 64.27 | 52.84 |
| Ovary-2 | 34715556 | 32784186 | 76.68 | 63.79 | 50.75 |
| Ovary-3 | 36697685 | 34654592 | 77.45 | 64.52 | 51.68 |
| Testis-1 | 38432756 | 36652050 | 77.31 | 64.12 | 51.86 |
| Testis-2 | 34641117 | 32964993 | 76.56 | 63.98 | 48.92 |
| Testis-3 | 37683948 | 35879253 | 77.21 | 64.2 | 51.84 |
